# Does Plasma Inhibit the Activity of KCl Cotransport in Red Cells From LK Sheep?

**DOI:** 10.3389/fphys.2022.904280

**Published:** 2022-05-24

**Authors:** David C.-Y. Lu, Anke Hannemann, John S. Gibson

**Affiliations:** Department of Veterinary Medicine, University of Cambridge, Cambridge, United Kingdom

**Keywords:** KCl cotransport, LK sheep, incubation media, volume, pH, urea

## Abstract

Red cells from LK sheep represent an important paradigm for control of KCl cotransport activity, as well as being important to sheep erythroid function. A previous report (Godart et al., 1997) suggested that autologous plasma markedly inhibits red cell KCC activity and identified the presence of the bicarbonate/CO_2_ buffer system as the probable cause. Findings were restricted, however, to red cells from patients with sickle cell disease (SCD) swollen anisotonically and carried out at a very high O_2_ tension (c.700 mmHg). It was therefore important to investigate the generality of the effect described and whether it was also relevant to the two main stimuli for KCC activity encountered most often by circulating red cells *in vivo* - low pH in active muscle beds during exercise and high urea concentrations in the renal medulla during antidiuresis. Results confirm that inhibition was significant in response to anisotonic swelling with KCC activity in MOPS-buffered saline (MBS) *vs*. bicarbonate-buffered saline (BBS) and in MBS *vs*. plasma both reduced (by about 25 and 50%, respectively). By contrast, however, inhibition was absent at low pH and in high concentrations of urea. These findings suggest therefore that red cell KCC activity represents an important membrane permeability *in vivo* in red cells suspended in plasma. They are relevant, in particular, to sheep red cells, and may also be important by extension to those of other species and to the abnormal red cells found in human patients with SCD.

## Introduction

It has become increasingly apparent that red cells are not simply quiescent carrier vehicles for haemoglobin (Hb) but rather have the ability to contribute to a rich complexity of responses. Physiologically, they participate in the overall homeostasis of organisms (eg from this journal: Pretini et al., 2019; Bogadnova and Kaestner, 2020), whilst, pathologically, their abnormal behaviour may contribute to a number of disease states (eg from elsewhere: Gallagher, 2017; Bernard, 2021). In humans, much evidence pertaining to the diversity of red cell function has been recently derived from a better appreciation of red cell phenotype reflects genotype in both healthy individuals and in patients with various disease states. By contrast, red cells from other animals have received relatively little attention.

Historically, comparative red cell research has provided an important resource for study. The behaviour of red cells from species across the animal kingdom—from lower vertebrates like *Amphiuma* to primates—illustrates the differing requirements of various species and how red cell function is adapted to their biology (Nikinmaa, 1990). Notable differences include the absence of band 3/anion exchanger in primitive fishes to the various energy sources for red cell glycolysis, as exemplified in mammals by the role of inosine (rather than glucose) in pig red cells, or by the presence of mitochondria in lower vertebrates, and to the way in which intracellular pH is regulated to modify Hb oxygen affinity.

A major transport function, Cl^−^-dependent cation transport, is present in the membranes of red cells from many different animals. Evidence for Cl^−^-cation cotransporters was first suggested in the late 1970s by work in Ehrlich ascites tumour cells (Hoffmann, 1978; Geck et al., 1980), and in low potassium-containing (LK) sheep red cells, and in humans (Dunham et al., 1980; Ellory and Dunham, 1980; Lauf and Theg, 1980). These systems are now established as protein-mediated coupled fluxes, as opposed to ones activated by the presence of Cl^−^. Three systems predominate: the first two, with Cl^−^ requiring the presence of both K^+^ and/or Na^+^, the Na^+^-K^+^-2Cl^−^ cotransporter (NKCC) and the Na^+^-Cl^−^ cotransporter (NCC); and, the third, a system dependent only K^+^ in addition to Cl^−^, the K^+^-Cl^−^ cotransporter (KCC). Their cloning and the establishment of the Cl^−^-cation cotransport family (SLC12A family including NCC; NKCC; KCC) has established that they function as true cotransport systems in numerous settings and many species (Herbert et al., 2004; Gamba, 2005).

Both KCC and NKCC, but not NCC, are widely present in red cells from different animals. They have a complex regulation which is often reciprocal but which is still incompletely understood. Physiological modulators include cell volume (Ellory and Dunham, 1980; Dunham and Ellory, 1981), intracellular pH (Ellory et al., 1991), intracellular [Cl^−^] (Voets et al., 1999), extracellular [K^+^] and [Cl^−^] (Lauf et al., 1992), urea (Dunham, 1995; Kaji and Gasson, 1995; Speake and Gibson, 1997), temperature (Khan and Ellory, 2000) and O_2_ tension (Borgese et al., 1991; Nielsen et al., 1992; Campbell and Gibson, 1998; Gibson et al., 1998; Gibson et al., 2000). Using protein kinase and phosphatase inhibitors of differing degrees of specificity (such as okadaic acid, calyculin, staurosporine, genistein, imantanib, etc), pharmacological studies have shown an important role for protein phosphorylation (Jennings and Al-Rohil, 1990; Jennings and Schulz, 1991; Starke and Jennings, 1993; Cossins et al., 1994; Flatman et al., 1996; Bize et al., 1999; Bize et al., 2000; Campbell et al., 2000).

Over the years, the behaviour of KCC in LK sheep red cells has provided an important paradigm for that in human red cells and in other species (eg Ellory and Dunham, 1980; Lauf and Theg, 1980; Lauf et al., 1992; Dunham, 1995; Campbell and Gibson, 1998; Campbell et al., 2000), and remains a valuable model system as well as being important in sheep erythrogenesis (Lauf and Bauer, 1987). It is worth noting that the genes for KCC are well conserved between human and sheep (eg for KCC1/SLC12A4 - https://www.ncbi.nim.ni.gov/gene6560 &/gene443178) and that sheep red cells have a similar low oxygen affinity to sickle cells (Samaja and Gattioni, 1978; Correra et al., 1984), which may be relevant when considering an oxygen-sensitive membrane transport system. This report focuses on the effect of plasma, as almost exclusively, studies investigating red cell KCC have used artificial media—simple salt solutions with synthetic buffers like HEPES, TRIS and MOPS—in which to suspend red cells whilst measuring K^+^ transport. A paper published towards the end of the 1990s, however, provocatively suggested that more physiological solutions—a bicarbonate-buffered saline (BBS) mimicking plasma, or plasma itself—contain constituents which markedly inhibit red cell KCC activity when compared to that measured in simple artificial saline (Godart et al., 1997). Red cell swelling was used as the stimulus for KCC activity, with cells suspended in MOPS-buffered saline (MBS), BBS or autologous plasma. An inhibition of KCC activity of over 80%, elicited by the presence of bicarbonate/CO_2_, rather than by that of some other component of plasma. The authors provokingly considered whether high levels of KCC activity may actually occur in circulating red cells *in vivo* (Godart et al., 1997). Their report, however, focussed on abnormal red cells from patients with sickle cell disease—which have several differences to those from normal individuals, not least a high reticulocyte count - whilst only anisotonic swelling was tested—which is unlikely to occur to a significant extent *in vivo*—and, in addition, very high O_2_ tensions (about 700 mmHg O_2_) were used—which might be problematical in an O_2_-sensitive system (Gibson et al., 2000). The applicability of their findings more generally therefore warrants further scrutiny.

This report focuses on the role of plasma in modulation of KCC activity in normal red cells from healthy LK sheep. As well as using anisotonic swelling as a stimulus, low pH and high levels of urea were also tested, as these represent the main physiological stimuli *in vivo*. In addition, a more realistic value for O_2_ tension (100 mmHg), approximating the maximum normally encountered in the arterial system *in vivo*, was used. Results suggest that plasma or the bicarbonate/CO_2_ buffer system is not as inhibitory as proposed and that KCC activity is therefore relevant to the function of red cells *in vivo* in the circulation.

## Materials and Methods

### Materials

All chemicals and inhibitors came from Sigma-Aldrich (Poole, Dorset, United Kingdom) unless otherwise stated. ^86^Rb^+^ came from Perkin-Elmer (Beaconsfield, Bucks., United Kingdom). Nitrogen, oxygen and carbon dioxide were from BOC Ltd. (Guildford, Surrey, United Kingdom).

### Blood Samples K^+^ Concentrations and Red Cell Volume:

Sheep blood samples were obtained freshly from a commercial source (TCS Biosciences Ltd., Botolph Claydon, United Kingdom) using heparin as an anticoagulant. Samples were immediately refrigerated or kept on ice until use, within two days. Whole blood was centrifuged gently at 600 g and the plasma removed and also stored on ice. Red cells were then washed in MOPS-buffered saline (see below) to remove any residual plasma and the buffy coat. Washed red cells and cell-free plasma were then stored separately on ice. Red cell and plasma [K^+^]s were measured by The Queen’s Veterinary School Hospital Central Diagnostic Services, University of Cambridge. For this purpose, red cell aliquots were lysed in ten-times their own volume of distilled water. [K^+^] of red cells varied from 7 to 16 mM (12.8 ± 1.6, mean ± S.E.M., *n* = 11), confirming that they came from LK sheep; that of plasma varied from 4.1 to 5.1 mM (4.2 ± 0.2, mean ± S.E.M., *n* = 11) and was adjusted through addition of KNO_3_ (100 mM) to a final value of 7.5 mM. Red cell volume was assessed as water content following the method of Borgese et al. (1987) and expressed as ml water per g dry cell solids (ml.g^−1^ d.c.s.).

### Salines and Inhibitors

MOPS-buffered saline (MBS) comprised (in mM): NaCl 137.5, KNO_3_ 7.5, MOPS 10, glucose 5, pH 7.4 at 37°C. For bicarbonate-buffered saline (BBS), 24 mM NaCl was replaced with NaHCO_3_. Wash solution (W-MBS) for removing unincorporated radioisotope was isotonic MgCl_2_ solution: MgCl_2_ 107, MOPS 10, pH 7.4 at 0°C. All solutions had an osmolality of 290 mOsm.kg^−1^ and a pH of 7.4, unless the effect of anisotonicity or pH was being studied when media of pH 7.0 and 6.5 were also prepared, and the effect of added hypertonic sucrose and water tested. For MBS, pHs were adjusted through addition of NaOH or HNO_3_ (in the absence of CO_2_), whilst for BBS and plasma, the media was equilibrated with the requisite partial pressure of CO_2_ (see below).

### Tonometry

KCC activity in red cells from many species is O_2_-sensitive (Gibson et al., 2000). It was therefore important to regulate O_2_ tension during incubation. Cells were gently rotated at 37°C in Eschweiler tonometers, coupled to a Wösthoff gas mixing pump (Speake et al., 1997) to set the O_2_ tension at the requisite level, either 677 or 100 mmHg O_2_ by flushing with pre-warmed and humidified air or 100% O_2_ mixed with N_2_. When using BBS or plasma, the gas mixture also included CO_2_ at a partial pressure necessary to take the pH to 7.4, 7.0 or 6.5 (typically 43 mmHg, 100 mmHg or 321 mmHg CO_2_, respectively). Cell suspensions were placed in the tonometers at ten-fold the haematocrit (Hct) needed for transport assay and equilibrated with the requisite gas mixture. They were then diluted ten-fold into test tubes, also pre-equilibrated at the required O_2_ and CO_2_ levels. Tubes were also gassed during incubation, but not bubbled (to prevent red cell lysis). Fully humidified gases at 37°C were used, which is necessary to prevent dehydration of the samples and to prevent condensation. All glassware and tubing was submerged and kept at 37°C.

### Measurement of KCC Activity

^86^Rb^+^ was used as a K^+^ congener. After dilution of the red cell samples into the test tubes, the influx was started by addition of ^86^Rb^+^ (final activity 0.02–0.05 MBq.ml^−1^) in distilled water at a 1 in 20 dilution. The duration of uptake was 10 min—control experiments have established that uptake is linear over this time period—and determinations were carried out in triplicates or quadruplicates. Uptake was stopped by diluting aliquots of the cell suspension into ice-cold W-MBS. Previous work has shown that K^+^ uptake achieves the new steady state within a minute or so of altering media conditions and that this is maintained for the duration of the incubations described here (eg Jennings and Al-Rohil, 1990). Unincorporated ^86^Rb^+^ was removed by centrifugation (10 s at 15,000 g), aspiration of supernatant and addition of further wash solution (4 washes and 5 spins in total). After each centrifugation step, cells were resuspended by gentle vortexing. Following the final wash, the cell pellet was lysed with Triton X-100 (0.1%) and protein (mainly haemoglobin in the case of red cells) precipitated with trichloroacetic acid (TCA, 5%). A final centrifugation step was used to harvest the clear, colourless supernatant before counting Čerenkov radiation by liquid scintillation (Packard Tri-carb 2800TR). Ouabain (100 µM) and bumetanide (10 µM) were present during all transport assays to inhibit K^+^ uptake *via* the Na^+^/K^+^ pump and Na^+^-K^+^-2Cl^−^ cotransporter (NKCC), respectively. K^+^ influx was calculated in mmol K^+^(l cells.h)^−1^. Under the conditions used, Cl^−^-independent K^+^ influx or K^+^ influx *via* NKCC were minimal, < 0.1 mmol K^+^ (l cells.h)^−1^, less than 5% of the total influx, so that the values given are taken as measures of KCC activity.

### Statistics

Data are given as means ± S.E.M. for samples from n different sheep. K^+^ influxes at different tonicities, pHs or in the presence/absence of urea were all made simultaneously in paired samples from the same sheep, with red cells incubated in each of the three different media at the same time. Statistical comparisons were made using Student’s t test for paired samples and ANOVA where more than two conditions were compared. A value of *p* < 0.05 taken as significant.

## Results and Discussion

### Control Experiments: Cell Volume and O_2_ Tension

The activity of KCl cotransport (KCC) in red cells from LK sheep is markedly affected by pH, volume, O_2_ tension and temperature, as well as by the concentrations of extracellular K^+^ and Cl^−^. All of these were therefore controlled carefully. Red cell water contents in MOPS -buffered saline (MBS), bicarbonate-buffered saline (BBS) and plasma were 1.97 ± 0.04, 1.97 ± 0.04 and 1.97 ± 0.04 ml g^−1^ d.c.s. (N.S.), respectively, showing that cell volume was not affected by the media used. This finding is consistent with modulation by intracellular pH and anisotonicity having the main effects on red cell volume. KCC activity was also compared in the different media equilibrated with an O_2_ tension of either 100 mmHg O_2_ or 677 mmHg, the latter value used by Godart et al. (1997). The magnitude of fluxes was reduced by about 25% at the lower O_2_ tension—for example at pH 7.4 K^+^ influx reduced from 2.20 ± 0.24 mmol (l cells.h)^−1^ to 1.62 ± 0.32 (*n* = 3)—but otherwise the pattern of responses to different modalities of stimulus (volume, pH and urea) and the effect of the incubation in the two other incubation media, BBS or plasma, were similar and unaffected by the two O_2_ tensions tested. In the following, results are therefore shown only for an O_2_ of 100 mmHg.

### Effect of Anisotonicity on KCC Activity

**FIGURE 1:**
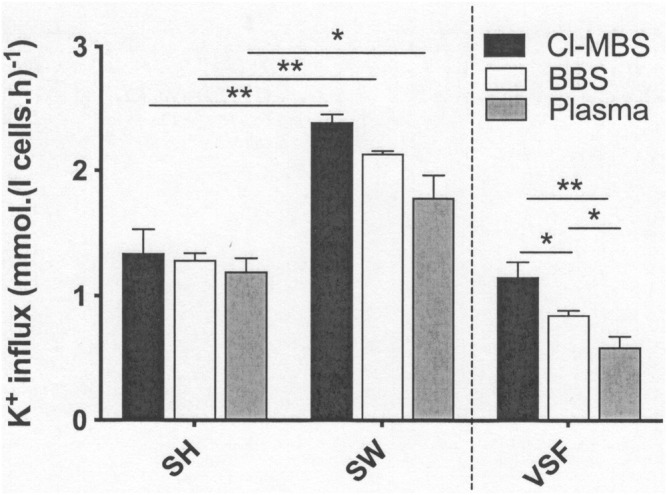
The effect of incubation media on volume-sensitive KCl cotransport activity in LK sheep red cells. Red cells (50% Hct) from LK sheep were equilibrated in Eschweiler tonometers for 15 min at an O_2_ tension of 100 mmHg under isotonic conditions (osmolality 290 mOsm.kg^−1^). Extracellular pH was pH 7.4, adjusted using HNO_3_/NaOH in MOPS-buffered saline (MBS), or with CO_2_ (43 mmHg) in the case of bicarbonate-buffered saline (BBS) and plasma. Red cell aliquots were then diluted ten-fold into test tubes pre-equilibrated with the same gas mixtures to alter cell volume anisotonically and for measurement of K^+^ influx (extracellular [K^+^] of 7.5 mM) over 10 min. Cell volume was altered through addition of hypertonic sucrose (15% shrunken; SH) or water (15% swollen; SW). All K^+^ influxes were carried out in the presence of ouabain (100 µM) and bumetanide (10 µM) to inhibit K^+^ influx through the Na^+^/K^+^ pump and Na^+^-K^+^-Cl^-^ cotransporter (NKCC), respectively. The volume-sensitive component of the K^+^ flux (VSF) represents the changes induced by altered (at ± 15% osmolality cf isotonic conditions). Histograms represent means ± S.E.M., *n* = 4. ∗*p* ≤ 0.05; ∗∗*p* ≤ 0.01.

The following series of experiments investigated the effects of incubation media on KCC activity altered through anisotonicity, with LK sheep red cells shrunken or swollen by 15% through addition of water or hypertonic sucrose (Figure 1). Cell volumes were not measured but previous work has established that alterations due to anisotonic media are complete. In all three media, anisotonic swelling significantly increased KCC activity by about 50%, consistent with previous reports (Dunham et al., 1980; Dunham and Ellory, 1981; Lauf and Adragna, 1992). KCC activity was highest in MBS, followed by BBS, and lowest in plasma. Fluxes in swollen red cells was reduced by about 10% comparing BBS with MBS, and by about 25% comparing plasma with MBS. Reduction in the component of KCC activity altered by aniostonicity, defined as the difference in K^+^ influx in red cells swollen compared with shrunken ones (= volume-sensitive K^+^ flux), was about 25% comparing BBS with MBS and about 50% comparing plasma with MBS (Figure 1). Although percentage inhibition was more modest in the bicarbonate-buffered media, these findings were similar to those described previously in red cells from SCD patients whose volume was altered similarly by an anisotonicity of 15% but at high O_2_ tension, about 700 mmHg (Godart et al., 1997), and in which the corresponding inhibition of volume-sensitive K^+^ fluxes was about 80% (MBS to BBS) and 75% (MBS to plasma). Inhibition was not due to altered red cell volume in the different media (Godart et al., 1997 and see above). In both the present work and that of Godart et al. (1997), K^+^ fluxes in shrunken red cells were unaffected by the medium used.

### Effect of pH on KCC Activity

**FIGURE 2:**
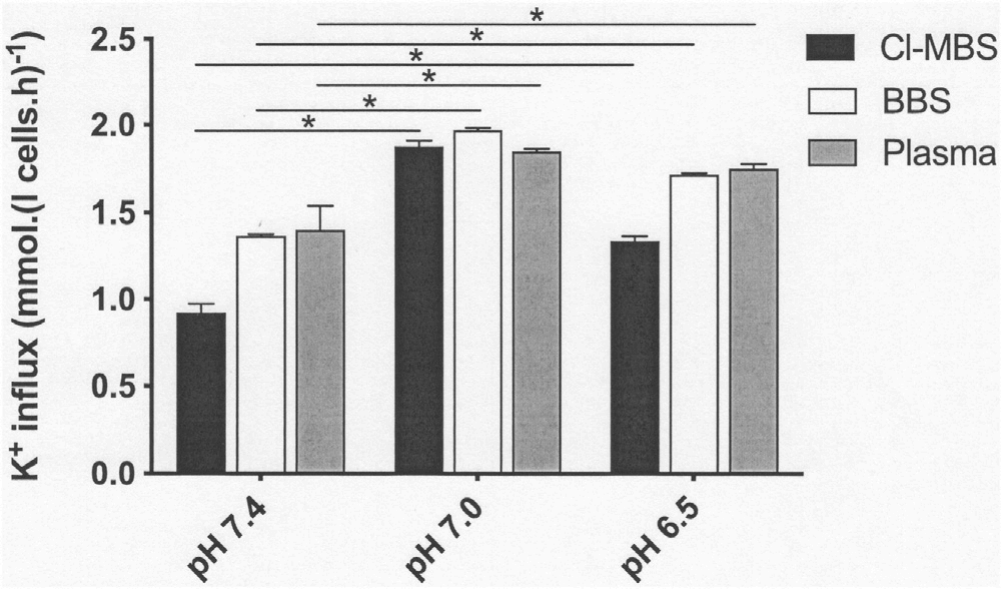
The effect of incubation media on pH-sensitive KCl cotransport activity in LK sheep red cells. Red cells (50% Hct) from LK sheep were equilibrated in Eschweiler tonometers for 15 min at an O_2_ tension of 100 mmHg under isotonic conditions (osmolality 290 mOsm.kg^−1^). The pHs of the incubation media were pH 7.4, pH 7.0 and pH 6.5, adjusted using HNO_3_/NaOH in MBS, but with CO_2_ in the case of BBS and plasma (with a CO_2_ of 43 mmHg at pH 7.4, 100 mmHg at pH 7.0 and 321 mmHg at pH 6). RBC aliquots were then diluted ten-fold into test tubes pre-equilibrated with the same gas mixtures for measurement of K^+^ influx (extracellular [K^+^] of 7.5 mM) over 10 min. All influxes were carried out in the presence of ouabain (100 µM) and bumetanide (10 µM) to inhibit K^+^ influx through the Na^+^/K^+^ pump and NKCC, respectively. Histograms represent means ± S.E.M., *n* = 3. ∗*p* ≤ 0.05.

The effect of altered extracellular pH was then examined (Figure 2). The response of LK sheep red cell KCC activity showed a bell-shaped response, as described previously (Lauf and Adragna, 1992), with increased activity as pH fell from pH 7.4 to pH 7.0, with a subsequent decrease as pH was further reduced to pH 6.5. At pH 7.0, KCC activity was not significantly different regardless of which incubation medium was used. At pH 7.4 and 6.5, activity was lowest in MBS, and no difference was observed in that measured in BBS or plasma. As a consequence, the pH-sensitive component of KCC activity (defined as the increase in K^+^ influx at pH 7.0 compared to that at pH 7.4) declined by about 40 and 50%, comparing MBS with BBS or plasma, respectively. The magnitude of the pH-sensitive component was not significantly altered, however, when comparing BBS and plasma (Figure 2). Overall, the reduction in pH-sensitive components of KCC activity in LK sheep red cells, by incubation in BBS or plasma compared with those measured in MBS, was due to the reduced activity measured in MBS at pH 7.4, and not due to decreased fluxes in BBS and plasma when measured at pH 7.0 or 6.5. In fact, the magnitude of KCC activity at these lower pHs was the same as in MBS or higher.

### Effect of Urea on KCC Activity

**FIGURE 3:**
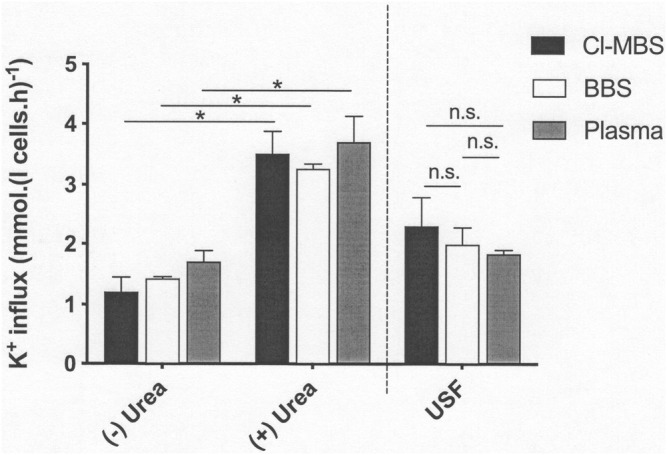
The effect of incubation media on urea-stimulated KCl cotransport activity in LK sheep red cells. Red cells (50% Hct) from LK sheep were equilibrated in Eschweiler tonometers for 15 min at an O_2_ tension of 100 mmHg under isotonic conditions (osmolality 290 mOsm.kg^−1^). Extracellular pH was pH 7.4, adjusted using HNO_3_/NaOH in MBS, or with CO_2_ (43 mmHg) in the case of BBS and plasma. Red cell aliquots were then diluted ten-fold into test tubes pre-equilibrated with the same gas mixtures for measurement of K^+^ influx (extracellular [K^+^] of 7.5 mM) over 10 min, in the continued absence of urea (-) or in its presence (+) at 750 mM. All influxes were carried out in the presence of ouabain (100 µM) and bumetanide (10 µM) to inhibit K^+^ influx through the Na^+^/K^+^ pump and NKCC, respectively. The urea-sensitive K^+^ flux (USF) represents the changes of flux induced by the presence of high urea concentrations (750 mM) compared with that measured in its absence. Histograms represent means ± S.E.M., *n* = 4. ∗*p* ≤ 0.05; n.s. indicates “not significant”.

In the last series of experiments, the stimulatory action of urea was investigated. Incubation at high concentrations of urea (750 mM) significantly increased KCC activity in all three media (Figure 3). In this case, the elevated values observed in the presence of urea were not significantly different in any of the media. Values measured in its absence at pH 7.4 were also similar across the three media, although the trend was for lower fluxes in MBS compared to BBS and plasma, similar to that observed in the previous experiment (Figure 2). The urea-stimulated component (defined as the increase in KCC activity in the presence of urea compared with that measured in its absence) was therefore also unaffected by the incubation media (Figure 3).

## Conclusion

In human red cells from patients with SCD, plasma markedly reduced KCC activity (Godart et al., 1997). This effect, however, was only determined in anisotonically swollen sickle cells. A careful analysis showed that inhibition was due predominantly to the presence of the physiological bicarbonate/CO_2_ buffer system, and not to physiological levels of Ca^2+^, Mg^2+^, phosphate or any alteration in cell volume between the different media. Both V_max_ and affinity (ie increased K_m_) were reduced. There was no such inhibitory action of plasma in red cells whose KCC activity was pharmacologically activated with protein kinase inhibitors, staurosporine and *N*-ethylmaleimide, indicating that the effect was likely *via* the regulatory phosphorylation cascade. The present results using red cells from LK sheep confirmed inhibition by bicarbonate/CO_2_ and plasma under anisotonic swelling. However, it was noticeable that stimulation by the two modalities most commonly encountered by circulating red cells *in vivo*, low pH and urea, was unaffected. We conclude that a significant inhibitory effect of the constituents of plasma may not be universal. This may be relevant to a role for red cell KCC in sheep and other species. For example, altered red cell pH is important for maternofetal oxygen transfer (Bunn, 1981; Nikinmaa, 1990). It may also be especially significant in human sickle cells in which hyperactivity of KCC is proposed to contribute significantly to red cell dehydration (Brugnara et al., 1986) and hence represents an important mechanism in pathogenesis of the disease. Finally, as the roles of other mechanosensitive transport systems notably PIEZO1 become clearer in healthy red cells and various disease states (eg Gallagher, 2017), it would be interesting to investigate some of the multiple modalities of stimulus to which KCC has been shown to respond (oxygen, pH, urea), and to also include any effect of incubation media on these other transporters (Bernhardt and Ellory, 2003)^1^.

## Data Availability

The raw data supporting the conclusion of this article will be made available by the authors, without undue reservation.

## Footnotes

1 Ncbi. https://www.ncbi.nim.ni.gov/gene6560 &/gene443178.
